# Influence of Abscisic Acid and Sucrose on Somatic Embryogenesis in Cactus *Copiapoa tenuissima* Ritt. forma *mostruosa*

**DOI:** 10.1155/2013/513985

**Published:** 2013-06-12

**Authors:** J. Lema-Rumińska, K. Goncerzewicz, M. Gabriel

**Affiliations:** Laboratory of Biotechnology, Department of Ornamental Plants and Vegetable Crops, University of Technology and Life Sciences in Bydgoszcz, Bernardyńska 6, 85-029 Bydgoszcz, Poland

## Abstract

Having produced the embryos of cactus *Copiapoa tenuissima* Ritt. forma * monstruosa* at the globular stage and callus, we investigated the effect of abscisic acid (ABA) in the following concentrations: 0, 0.1, 1, 10, and 100 **μ**M on successive stages of direct (DSE) and indirect somatic embryogenesis (ISE). In the indirect somatic embryogenesis process we also investigated a combined effect of ABA (0, 0.1, 1 **μ**M) and sucrose (1, 3, 5%). The results showed that a low concentration of ABA (0-1 **μ**M) stimulates the elongation of embryos at the globular stage and the number of correct embryos in direct somatic embryogenesis, while a high ABA concentration (10–100 **μ**M) results in growth inhibition and turgor pressure loss of somatic embryos. The indirect somatic embryogenesis study in this cactus suggests that lower ABA concentrations enhance the increase in calli fresh weight, while a high concentration of 10 **μ**M ABA or more changes calli color and decreases its proliferation rate. However, in the case of indirect somatic embryogenesis, ABA had no effect on the number of somatic embryos and their maturation. Nevertheless, we found a positive effect of sucrose concentration for both the number of somatic embryos and the increase in calli fresh weight.

## 1. Introduction

*Copiapoa tenuissima *Ritt. forma* mostruosa* is a member of the Cactaceae family, and it is a spontaneous mutant derived from *Copiapoa tenuissima *Ritt. (syn.* Neochilenia wageringeliana*) species [1]. This species originated in extremely dry desert areas of Chile, and its name has been linked to the town of Copiapo where the small number of its natural habitats exists [2]. Currently, this species is strictly protected (entered into Appendix II Cactaceae species of the CITES 1997). *C. tenuissima *Ritt. forma *mostruosa*, which differs from the original species in the lack of thorns and rare, almost black epidermis color, is one of the most interesting cactus species most desired by collectors globally. Its attractive appearance is additionally enhanced by white wool-like areoles. To prove an effective protection to the natural habitats of this cactus, it is necessary to develop effective propagation methods under artificial conditions. Under natural conditions the formation of new axillary shoots is slow; shoot growth by the activation of meristems in areoles can be much faster [3]. But the most effective methods of micropropagation are based on somatic embryogenesis which allows for producing valuable genotypes on a large scale in a very short time. Somatic embryogenesis may be induced via a direct or indirect pathway. For direct somatic embryogenesis, embryos develop directly on the surface of organized tissue. Alternatively, indirect somatic embryogenesis may occur via an intermediate step involving callus formation. Both the direct and indirect somatic embryogenesis make the regeneration of plants from single somatic cells possible [4]. Minocha and Mehra [5] reported the first regeneration of somatic embryos in cactus *Neomammillaria prolifera*. Since then, many applicable reports on cacti have been published [6–10], but only one on *Copiapoa* genus [11]. A critical stage of somatic embryogenesis is the maturation stage when embryos accumulate up storage materials [12, 13]. This stage depends on the presence of specific plant growth regulators (PGRs), mostly abscisic acid (ABA) and sucrose [14–16]. ABA increases the level of storage proteins and fatty acids in somatic embryos [15–17]. Abscisic acid plays a significant role in the regulation of many physiological processes of plants. It is often used in tissue culture systems to promote somatic embryogenesis and enhance somatic embryo quality by increasing desiccation tolerance and preventing precocious germination [18]. Sucrose, as a source of energy and carbon skeletons, determines the growth potential of the plant [19] and also affects the quality of embryos [15].

The aim of the present study was to determine the effect of ABA and sucrose on direct and indirect somatic embryogenesis in cactus *Copiapoa tenuissima *Ritt. f. * mostruosa.*

## 2. Materials and Methods

Plant materials were mammillae of cacti *Copiapoa tenuissima* Ritt. forma *mostruosa*. The cactus was grafted onto the pad (stem) from the genus *Cereus. *The initial explants (400 mammillae with areoles) were taken from the central zones of donor plants (average height: 6 cm) from the collection of Licznerski (Jarużyn Kolonia near Bydgoszcz, Poland).

### 2.1. Direct Somatic Embryogenesis (DSE)

#### 2.1.1. Induction Stage

The explants were surface disinfected with 70% ethanol for 1-2 s and then with 0.79% hypochloride solution for 15 min, followed by three rinses with distilled sterilized water (all steps in laminar flow cabinet). Then they were cultured (one explant per jar) on MS [20] basal salts medium with additional 1506.2 *μ*M CaCl_2_·6H_2_O, 50.0 *μ*M FeSO_4_·7H_2_O, and 55.3 *μ*M Na_2_EDTA·2H_2_O. The medium contained 3% sucrose, solidified with 1.2% Purified Lab Agar (Biocorp); the media pH was adjusted to 5.7 prior to autoclaving. The explants were cultured on MS medium with 9.05 *μ*M auxin 2,4-D (2,4-dichlorophenoxyacetic acid) or MS medium without PGRs (as control). The cultures were kept in a growth room at 24 ± 2°C and exposed to 16 h photoperiod. Daylight was maintained by using Philips TLD 54/34 W lamps with a photon flux density of 40.4 *μ*mol·m^−2^·s^−1^. Induction stage developed during 8 weeks. 

#### 2.1.2. Influence of ABA on DSE

At the successive stage of the experiment, randomly selected mammillae with somatic embryos were transferred onto 5 types of modified MS media containing ABA at different concentrations: 0; 0.1; 1; 10; 100 *μ*M. Each type of media was represented by 12 explants with four replications (3 explants per jar). Other medium components and *in vitro* culture conditions were identical to the ones described previously in induction stage. After 6 weeks of culture the analysis of somatic embryos was made using the stereomicroscope.

### 2.2. Indirect Somatic Embryogenesis (ISE)

#### 2.2.1. Induction Calli Stage

Calli were obtained on a modified MS medium containing 9.05 *μ*M 2,4-D from initial explants (mammillae with areoles). The successive calli-proliferating transfers were made regularly every 3 weeks on a modified MS medium supplemented with 13.32 *μ*M BA, 16.11 *μ*M NAA, and 0.57 *μ*M IAA added to produce an adequate amount of callus for further research. The other medium components and culture conditions were the same as described for the induction stage of the direct somatic embryogenesis induction. The calli were yellow-green in color and demonstrated strong proliferation properties.

#### 2.2.2. Effect of ABA and ABA and Sucrose

The calli were divided into fragments and the initial fresh weight was registered. Next, they were cultured onto modified MS media containing ABA at different concentrations (0; 0.1; 1; 10; 100 *μ*M) or onto modified MS media with ABA (0; 0.1; 1 *μ*M) and sucrose (1; 3; 5% w/v). Despite different concentrations of ABA or ABA and sucrose, the MS media contained a fixed number of PGRs facilitating calli proliferation (13.32 *μ*M BA, 16.11 *μ*M NAA, and 0.57 *μ*M IAA). After 5 weeks the calli fresh weight was registered again, and the embryo structures were analyzed under the stereomicroscope. 

### 2.3. Statistical Analysis

The experiments were arranged in a completely randomized design with four replicates per treatment. Each type of media was represented by 12 explants each; four replicates (3 explants per jar). The data were evaluated by analysis of variance, and comparisons between the mean values were made by the *t*-Student test at *α* = 0.05.

## 3. Results

### 3.1. Direct Somatic Embryogenesis

**Figure 1:**
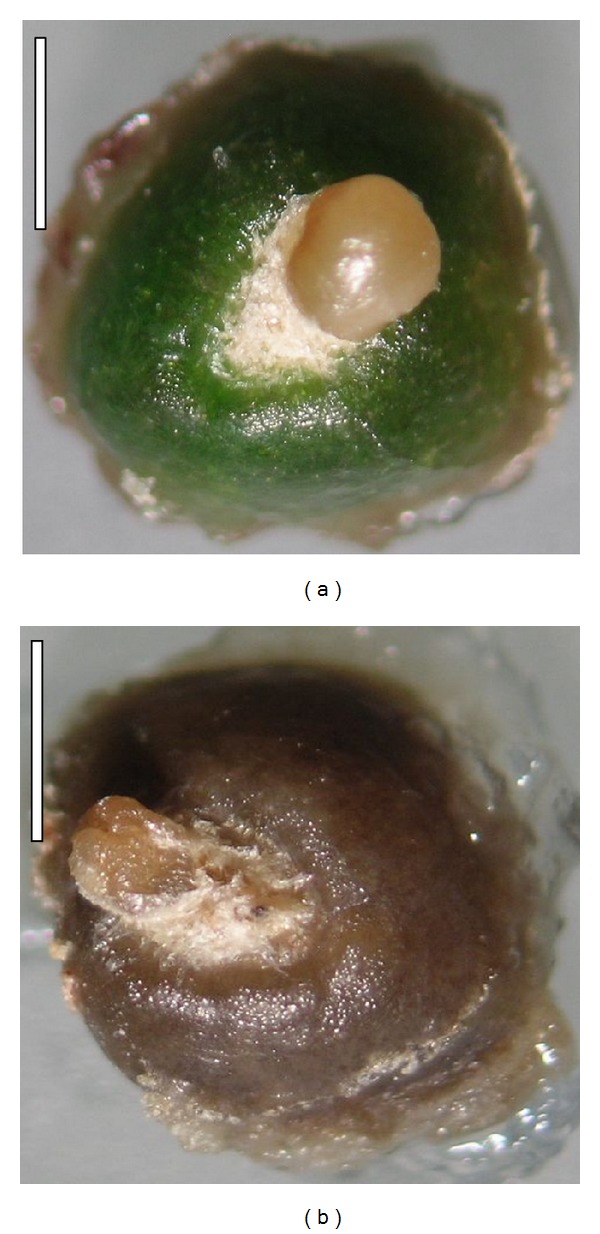
Somatic embryos on mammillae of the cactus *C. tenuissima* Ritt. forma* mostruosa* developed on the medium (bar = 1 mm): (a) without ABA and (b) with a high concentration of ABA (10 *μ*M).

**Figure 2:**
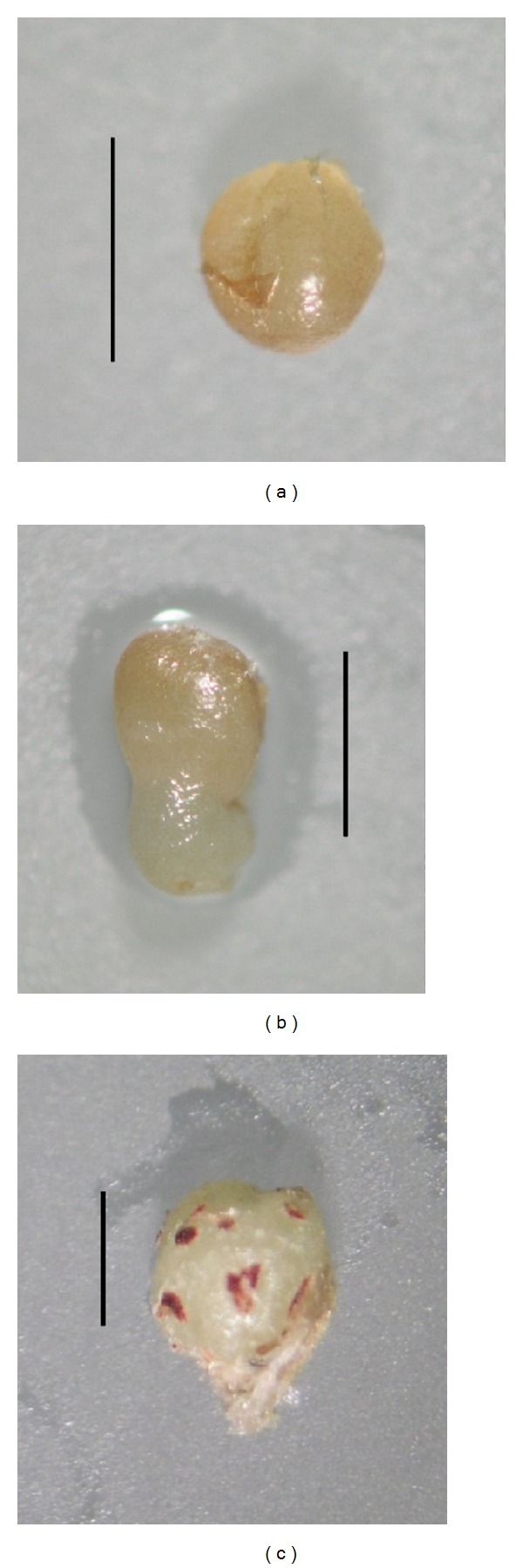
Developmental stages observed in somatic embryos obtained in direct somatic embryogenesis in the cactus *C. tenuissima* Ritt. forma* mostruosa *(bar = 1 mm): (a) globular, (b) torpedo, and (c) shoot developed from somatic embryo.

During induction stage, we observed the regeneration of somatic embryos at the globular stage on 20.67% of mammillae cultured in media containing 9.05 *μ*M 2,4-D. The embryos were cream yellow in color. Bacterial and fungal contamination accounted for 12.67%. Next, at the stage 2, we transferred the mammillae with somatic embryos at the globular stage onto media supplemented with different ABA concentrations, and we found that further elongation growth of embryos occurred only on the media with a low ABA content (Table 1). The media with a high concentration of this PGR (10 and 100 *μ*M) inhibited the growth of embryos and resulted in an evident color change in embryos from cream-yellow to brown (Figures 1(a) and 1(b)). On media with a low content of ABA (0-1 *μ*M) the embryos were round in shape and their epidermis was shiny, while mammillae remained green. Also, we observed three developmental stages: globular, torpedo, and shoot (Figures 2(a), 2(b), and 2(c)). The globular stage that predominated on all the media was evaluated accounting for 84.6 to 91.7%. Only on MS ABA0.1 and MS ABA1 torpedo stages were able to develop. However, on MSABA0.1 the embryo developed into a spherical shaped shoot white in color with maroon points being the developing areoles (Figure 2(c)). The proximal part of the shoot was visibly becoming green. In this medium we also observed secondary somatic embryogenesis.

### 3.2. Indirect Somatic Embryogenesis

**Figure 3:**
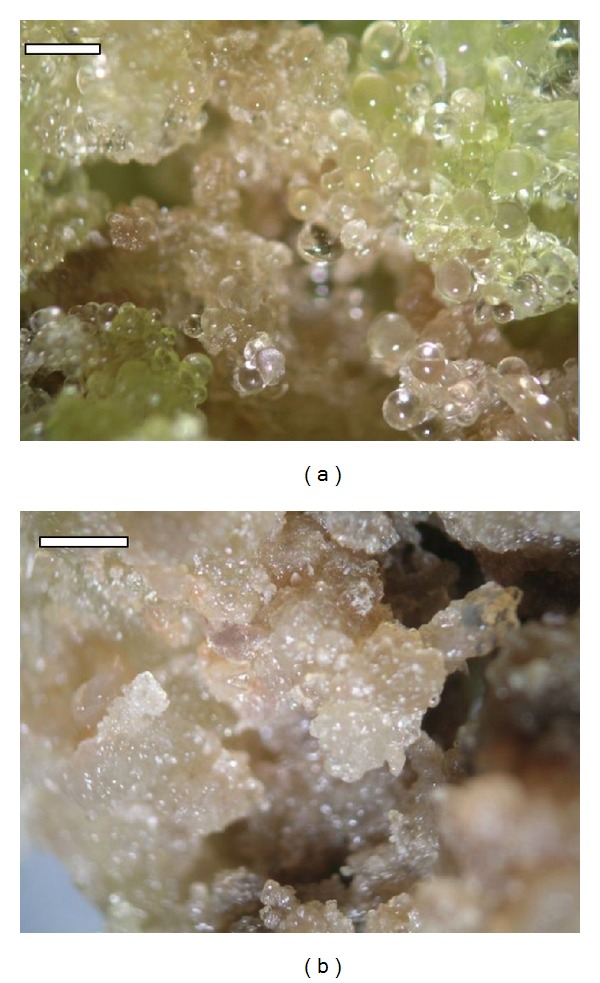
Callus of the cactus *C. tenuissima* Ritt. forma *mostruosa* on the media (bar = 0.1 mm): (a) without ABA (magnification 1.5 × 10) and (b) with a high concentration of ABA (10 *μ*M; magnification 1.4 × 10).

 In experiment 1, media with low ABA concentration (0-1 *μ*M), we noted a more-intensive growth of the fresh weight of callus than on the media of a high ABA concentration (10–100 *μ*M) (Table 2). Additionally, a high concentration of ABA was noted to coincide with changes in calli structure and color from yellow into cream and cream-brown (Figures 3(a) and 3(b)). However, the ABA concentration did not have a significant effect on the number of somatic embryos produced and their development stages (Table 3). Three development stages were found: globular, heart, and torpedo; most embryos, however, were isolated at the globular stage on the media with 0 and 1 *μ*M ABA, where they accounted for 100% of all the embryos. Further development stages, namely, heart, occurred at 10 and 100 *μ*M ABA, while torpedo stage occurred at 0.1, 10, and 100 *μ*M ABA. The results observed in experiment 2, investigating the effect of ABA and sucrose, coincided with the results of experiment 1. Here, we identified no effect of ABA on the number of somatic embryos regenerated and their degree of maturity (Table 4). However, we noted a significant effect of the sucrose concentration on the number of somatic embryos. The lowest number of embryos was produced at 1% concentration, while at 3 and 5% a significantly higher number of somatic embryos resulted (Table 4). Similarly an increase in the calli fresh weight was greater at sucrose concentrations of 3 and 5%.

## 4. Discussion

In the cactus *Copiapoa tenuissima *Ritt. forma* mostruosa* auxin 2,4-D plays a decisive role in the induction of somatic embryogenesis both directly and indirectly. Similar to many other plant species, this process is induced by exogenous 2,4-D [10, 21–23]. In the cactus investigated, 9.05 *μ*M 2,4-D resulted in the production of 20.67% explants regenerating embryos at the globular stage and 5.67% explants regenerating calli. Also, secondary somatic embryogenesis was observed on young shoots that were developed on mammillae of the studied cactus. Secondary embryogenesis has been reported for several plant species as coronation [24], peanut [25], *Medicago truncatula* [26], and *Helianthus maximiliani* [27] cultured on media containing variation types of auxins, particularly 2,4-D. Further somatic embryo development stages occur very often after the elimination of auxin from the medium and adding PGRs favourable to somatic embryo maturation. Among the PGRs most frequently applied in the process of somatic embryo maturation is ABA [28–30]. Cardoza and D'Souza [29] reported the maturation of somatic embryos from the globular to heart and cotyledon stages in *Anacardium occidental* L. on the MS medium containing 2 *μ*M ABA. In the present study a low ABA concentration (0-1 *μ*M) stimulated the elongation of embryos at the globular stage and the number of adequate embryos in direct somatic embryogenesis, while a high ABA concentration (10–100 *μ*M) resulted in growth inhibition, and on somatic embryos wrinkles formed as well as the primary explants, mammillae turning brown. A low concentration of ABA (2.5–7.5 *μ*M) also enhanced the development of adequately developed somatic embryos in *Cocos nucifera* L. [21]. However, as reported by Cailloux et al. [14] in *Hevea brasiliensis*, a high ABA concentration (10 *μ*M) combined with a high concentration of sucrose enhances the maturation of embryos. Baskaran and Van Staden [31] showed that the addition of abscisic acid (1.9 *μ*M) to the medium significantly improved the development of somatic embryos and their conversion to plantlets in *Merwilla plumbea* (Lindl.) Speta. Our results also suggest that the application of low ABA concentrations (0.1–1 *μ*M) is favourable to the maturation of somatic embryos in *Copiapoa*. Similarly, the study of ISE in this cactus suggests that lower ABA concentrations enhance the development of calli fresh weight, while at higher concentrations (10 *μ*M ABA or more) calli color changes and its proliferation rate decreases. Nevertheless, in the ISE no effect of ABA on the number of somatic embryos and their maturation was shown.   Similarly, Agarwal et al. [32] reported a negative effect of ABA with an increase in its concentration on *Morus alba* L. and a total inhibition of embryogenesis at the concentration of 10 *μ*M. Another essential factor  which facilitates the maturation of somatic embryos is sucrose. We identified a positive effect of sucrose (3 and 5%) both on the number of somatic embryos and on an increased calli fresh weight of *Copiapoa*. The application of a high concentration of sucrose (6%) definitely increases the size of somatic embryos in *Juglans regia* L. [30]. Similarly the reports by Agarwal et al. [32] point to a considerable role of sucrose (6%) in the process of somatic embryogenesis in *Morus alba* L, while Nakagawa et al. [16] reported that sucrose induces the somatic embryogenesis in melon (*Cucumis melo* L.). A negative effect on the development of cotyledonary in *Merwilla plumbea* was observed by Baskaran and Van Staden [31] with reduction of sucrose (below 3%) in the medium. On the other hand, Charrière and Hahne [33] found that the concentration of sucrose (3 or 12%) had a significant effect on the pattern of organogenesis at a low concentration of stimulated somatic embryogenesis at a higher concentration in sunflower (*Helianthus annuus* L.), whereas Sghaier et al. [17] showed that both a high sucrose concentration (9%) and a high ABA concentration (20 *μ*M) affect the morphology, rate of germination, and the content of storage protein in somatic embryos in date palm (*Phoenix dactylifera* L.).

## 5. Conclusions

We investigated the effect of abscisic acid (ABA) and sucrose on successive stages of DSE and ISE in cactus *Copiapoa tenuissima *Ritt. forma* mostruosa*. The results showed that a low concentration of ABA (0-1 *μ*M) stimulates the elongation of embryos, while the high ABA concentration (10–100 *μ*M) results in growth inhibition. The ISE study suggests that the lower ABA concentration enhances the increase in calli fresh weight, while the high concentration changes calli color and decreases its proliferation rate. The positive effect of sucrose concentration (3 and 5%) for both the number of somatic embryos and the increase in calli fresh weight was also observed.

## Figures and Tables

**Table 1:** The influence of ABA on direct somatic embryogenesis in cactus *C. tenuissima* Ritt. forma* monstruosa*.

				Somatic embryo				Stage of somatic embryo					
Medium	ABA (*μ*M)	Length of embryo	Color of embryo	Correct		Formed wrinkles		Globular		Torpedo		Shoot	
				Number	%	Number	%	Number	%	Number	%	Number	%
MS 0ABA	0	0.95 a*	Cream-yellow	11 a	91.7	1 b	8.3	11 a	91.7	1 a	8.3	0 a	0.0
MS 0.1ABA	0.1	0.97 a	Cream-yellow	20 a	100.0	0 b	0.0	17 a	85.0	2 a	10.0	1 a	5.0
MS 1ABA	1	0.90 a	Cream	11 a	91.7	1 b	8.3	11 a	84.6	2 a	15.4	0 a	0.0
MS 10ABA	10	0.80 b	Brown	3 b	25.0	9 a	75.0	11 a	91.7	1 a	8.3	0 a	0.0
MS 100ABA	100	0.80 b	Brown	3 b	25.0	9 a	75.0	11 a	91.7	1 a	8.3	0 a	0.0

*Data in columns marked with the same letter do not differ significantly at *α* = 0.05.

**Table 2:** Effect of ABA on calli fresh weight increase and percentage of somatic embryos regenerated from calli during indirect somatic embryogenesis of *C. tenuissima* Ritt. forma* monstruosa*.

Medium	ABA (*μ*M)	Calli fresh weight increase (g)	Calli with somatic embryos (%)
MS ABA0^1^	0	8.36 a*	66.7
MS ABA0.1	0.1	8.75 a	83.3
MS ABA1	1	7.29 a	66.7
MS ABA10	10	4.02 a	83.3
MS ABA100	100	0.50 b	66.7

^1^Except ABA, all media contained constant concentration of the following PGRs: 13.32 *μ*M BA; 16.11 *μ*M NAA; 0.57 *μ*M IAA.

*Data in columns marked with the same letter do not differ significantly at *α* = 0.05.

**Table 3:** Effect of ABA concentration on the number and the developmental stage observed in somatic embryos obtained during indirect somatic embryogenesis of *C. tenuissima* Ritt. forma* monstruosa*.

Medium	ABA (*μ*M)	Number of somatic embryos		Stage of somatic embryo					
		Total	On one cultured callus	Globular		Heart		Torpedo	
				Total	On one cultured callus	Total	On one cultured callus	Total	On one cultured callus
MS ABA0^1^	0	11	0.9 a	11	0.9 a	0	0.0 a	0	0.0 a
MS ABA0.1	0.1	21	1.8 a	20	1.7 a	0	0.0 a	1	0.1 a
MS ABA1	1	12	1.0 a	12	1.0 a	0	0.0 a	0	0.0 a
MS ABA10	10	35	2.9 a	33	2.8 a	1	0.1 a	1	0.1 a
MS ABA100	100	13	1.1 a	10	0.8 a	1	0.1 a	2	0.2 a

Explanation as in Table 2.

**Table 4:** Influence of ABA and sucrose contents on indirect somatic embryogenesis of *C. tenuissima* Ritt. forma*monstruosa*.

Medium	ABA (*μ*M)	Sucrose (%)	Calli fresh weight increase (g)	Calli with somatic embryo (%)	Number of somatic embryos	
					Total	On one cultured callus
MS 0ABA S1^1^	0	1	2.12 aB*	75	25	2.3 aB
MS 0ABA S3	0	3	8.94 aA	100	195	17.7 aA
MS 0ABA S5	0	5	10.57 aA	100	202	18.4 aA
MS 0.1ABA S1	0.1	1	1.45 aB	92	49	4.5 aB
MS 0.1ABA S3	0.1	3	10.70 aA	100	173	15.7 aA
MS 0.1ABA S5	0.1	5	10.76 aA	100	250	22.7 aA
MS 1ABA S1	1	1	0.96 aB	100	94	8.5 aB
MS 1ABA S3	1	3	6.48 aA	100	237	21.5 aA
MS 1ABA S5	1	5	10.14 aA	100	258	23.5 aA

^1^Except ABA and sucrose (S), all media contained constant concentration of the following PGRs: 13.32 *μ*M BA; 16.11 *μ*M NAA; 0.57 *μ*M IAA.

*a: data in columns marked the influence of ABA with the same lowercase letter do not differ significantly at *α* = 0.05.

A: data in columns marked the influence of sucrose with the same uppercase letter do not differ significantly at *α* = 0.05.

## Abbreviations

ABA: Abscisic acid
BA: 6-Benzylaminopurine
2,4-D: 2,4-Dichlorophenoxyacetic acid
DSE: Direct somatic embryogenesis
IAA: 3-Indolylacetic acid
ISE: Indirect somatic embryogenesis
MS: Murashige and Skoog medium
NAA: 1-Naphthylacetic acid.
